# FANCM-associated proteins MHF1 and MHF2, but not the other Fanconi anemia factors, limit meiotic crossovers

**DOI:** 10.1093/nar/gku614

**Published:** 2014-07-18

**Authors:** Chloe Girard, Wayne Crismani, Nicole Froger, Julien Mazel, Afef Lemhemdi, Christine Horlow, Raphael Mercier

**Affiliations:** 1 INRA, Institut Jean-Pierre Bourgin, UMR1318, ERL CNRS 3559,Saclay Plant Sciences, RD10, 78000 Versailles, France; 2 AgroParisTech, Institut Jean-Pierre Bourgin, UMR 1318, ERL CNRS 3559, Saclay Plant Sciences,RD10, 78000 Versailles, France

## Abstract

Genetic recombination is important for generating diversity and to ensure faithful segregation of chromosomes at meiosis. However, few crossovers (COs) are formed per meiosis despite an excess of DNA double-strand break precursors. This reflects the existence of active mechanisms that limit CO formation. We previously showed that *AtFANCM* is a meiotic anti-CO factor. The same genetic screen now identified *AtMHF2* as another player of the same anti-CO pathway. FANCM and MHF2 are both Fanconi Anemia (FA) associated proteins, prompting us to test the other FA genes conserved in Arabidopsis for a role in CO control at meiosis. This revealed that among the FA proteins tested, only FANCM and its two DNA-binding co-factors MHF1 and MHF2 limit CO formation at meiosis.

## INTRODUCTION

One prominent feature of eukaryotic sexual reproduction is meiosis, a specific type of cell division where two rounds of chromosome segregation follow a single round of DNA replication. This produces haploid spores from a diploid mother cell. At the first division, correct chromosome segregation relies on physical connections between homologues which are provided by crossovers (COs). COs are reciprocal exchanges of genetic material between homologues. These events are initiated by the formation of DNA double-strand breaks (DSBs) which will be repaired by homologous recombination as COs or non-crossovers (NCOs). At least two pathways to CO formation exist with different genetic requirements. Species exist with only one of these pathways; however Arabidopsis, humans and budding yeast, for example, have both (1). The first pathway, which is prominent in most species, is dependent on a group of proteins collectively referred to as ZMMs (for Zip1, Zip2, Zip3 and Zip4, Mer3 and Msh4–Msh5) and on the Mlh1–Mlh3 heterodimer, first identified in *Saccharomyces cerevisiae* and conserved in a large range of eukaryotes (2,3). The COs that arise from this pathway are sensitive to a phenomenon known as CO interference where one CO reduces the probability of another CO occurring at adjacent loci (4). The second pathway of CO formation involves the endonuclease MUS81 and produces COs that are not sensitive to interference (1). Interestingly, CO number is relatively low in most eukaryotes, being very close to the one, obligatory, CO per chromosome pair, despite a large excess of recombination precursors (5). This suggests that active mechanisms limit CO frequency, whose molecular factors remain largely unknown. The helicase Fanconi Anemia Complementation Group M (FANCM) has been found to be a major meiotic anti-CO factor in *Arabidopsis*, limiting MUS81-dependent CO formation, a normally minor pathway of CO formation in *Arabidopsis thaliana* (6). This function seems to be evolutionarily conserved as Fml1, the fission yeast FANCM ortholog, also directs NCO formation (7).

Fanconi Anemia (FA) is a rare heritable human disease that is characterized by early onset of bone marrow failure and susceptibility to certain cancers. The FA pathway, which implicates at least 16 proteins in human cells, appears to be present in all eukaryotes and promotes genome stability by resolving blocked replication forks (8,9). The FA genes have been initially identified as preventing FA in humans. The FA proteins can be categorized into three groups, according to their biochemical function. (i) The core complex is the first recruited to DNA stalled replication forks, using FANCM as a landing pad. Two newly discovered co-factors of FANCM, namely MHF1 and MHF2, have been shown to stimulate FANCM DNA-binding activity and its targeting to chromatin (10,11). (ii) The FA-ID complex is recruited and ubiquitinated by the core complex at the damage site. (iii) The downstream partners are thought to act independently of the first two groups but have strong links with the homologous recombination machinery, and mutation of any leads to development of the disease in human (8). Implication of FANCM in the control of meiotic CO formation raises the question whether other FA proteins limit meiotic COs, or if the FANCM meiotic function is unique among FA proteins. Here, using both forward and reverse genetic screens, we show that from a series of FA proteins conserved in Arabidopsis, only *At*MHF1 and *At*MHF2 were identified as CO-limiting factors. We propose that FANCM and its direct DNA-binding cofactors MHF1 and MHF2 prevent meiotic CO formation, without the other FA proteins being involved.

## MATERIALS AND METHODS

### FA protein identification

Homologues and putative homologues of FA-associated genes were identified using literature searches and reciprocal BLASTp and PSI-BLAST (http://www.ncbi.nlm.nih.gov/, http://www.arabidopsis.org/ and http://bioinformatics.psb.ugent.be/plaza).

### Genetic material

The lines used in this study were *Atmhf1–3* (N576310), *Atfanci* (N555483), *Atfancd2* (N613293), *Atfance* (N553587), *Atfancl* (37079— identified from the Max-Planck Institute für Züchtungsforschung collection from Köln, Germany (12)), *zip4-1* (EJD21) (13), *zip4-2* (N568052) (13), *shoc1-1* (N557589) (14), *msh5-2* (N526553) (15), *mus81-2* (N607515) (16), *spo11-1-3* (N646172) (17), *fancm-1* (6), *hei10-2* (N514624) (18), fluorescent-tagged lines (FTLs) I2ab (FTL1506/FTL1524/FTL965/*qrt1-2*) (19). Genotyping by polymerase chain reaction was performed with two primer pairs. The first pair is specific to the wild-type allele, and the second pair is specific to the left border of the inserted sequence as follows: *Atmhf1-3* (N576310U 5′-CCTAAACC-ATCCTCCAGCTTC-3′ and N576310L 5′-CAATTTAAAGACGCAGGATCG-3′, N576310L and LBSalk2 5′-GCTTTCTTCCCTTCCTTTCTC-3′); *Atfanci* (N555483U 5′-AGTCCAACACATGTCCTCCAC-3′ and N555483L 5′-TGAGTTTGGTGATTCGAAAGG-3′, N555483L and LBSalk2); *Atfancd2* (N613293U 5′-AATTCACCGGAATGTCACAAC-3′ and N613293L 5′-AATTCACCGGAATGTCACAAC-3′, and N613293L and LBSalk2); *Atfance* (N553587U 5′- TCAGCTGATGAAGACAGCATG-3′ and N553587L 5′-ATGTCAACCCACAGAGGATTG-3′, and N553587L and LBSalk2); *Atfancl* (FANCL-U 5′-ACAGAGATAAGAAGGGAAGAG-3′ and FANCL-L ATTATCATTAACCCGTCATTC, and FANCL-L and LB Gabi o8409 5′-ATATTGACCATCATACTCATTGC-3′). *mhf2* alleles were genotyped by dCAPS as follows: *mhf2-1* locus amplification with 5′-ATCTGCGAGCTTTTTTATTCGATTGCGATGAA-3′ and 5′-AGGAGTTACGATACCAAATGA-3′, subsequent digestion by MboII (104+33 bp for the wild-type amplicon and 137 bp for the mutant); *mhf2-2* locus amplification with 5′-AAGCGTTTATGTATTTTTAGA-3′ and 5′-CTTCTGGTTCGTTTATACACT-3′, subsequent digestion with BseNI (350 bp for the wild-type and 330+20 for the mutant).

*Atzip4(s)2* (*Atmhf2-1)* was sequenced using Illumina technology. Mutations were identified through MutDetect pipeline developed by Bioinformatics and Informatics IJPB team (Supplementary Methods).

### Cytology

Meiotic chromosome spreads have been performed as described previously (20). Immuno-localizations were performed as described in (21). Observations were made using a ZEISS AxioObserver microscope.

## RESULTS AND DISCUSSION

### *zmm* suppressor screens identified MHF2 as an anti-CO factor

We sought to find *Arabidopsis* mutants with increased CO-formation. However, increased meiotic CO formation does not confer any obvious macroscopic phenotype preventing easy genetic screening (6). In contrast, reduction in CO formation is easily detectable because without the physical connection provided by CO, pairs of homologous chromosomes do not associate as bivalents at metaphase I and appear cytologically as univalents that segregate randomly at anaphase I. At the macroscopic level, this lack of CO is reflected by reduced fertility easily noticed by shorter fruit. For instance, *zmm* mutants show a 75% reduction in bivalent formation and are almost sterile (2). Here we continue a previously described genetic screen, based on the idea that mutations increasing CO frequency will restore the fidelity of chromosome segregation and subsequently restore the fertility of *zmm* mutants (6). We continued the *Atzip4* (13) suppressor screen that previously revealed *AtFANCM* as an anti-CO gene. Among 2000 lines screened, eight recessive suppressors were found, falling into three complementation groups, the first of which corresponding to *FANCM* (6). The second complementation group contained one line, *zip4 suppressor 2* (*zip4(s)2*), and is the focus of this study. Map-based cloning defined a region between 27.15 Mb and 30.29 Mb on chromosome 1 as containing the causal mutation. Following whole genome sequencing, we identified a candidate mutation in the splice donor site of exon 2 in the gene *At1g78790*. In parallel, we ran a second screen looking for suppressors of another *zmm* mutant, *Athei10* (18). Among 2000 lines screened, 19 suppressors were found. Systematic sequencing of *At1g78790* in the suppressors revealed that two lines (*hei10(s)174* and *hei10(s)170*) also contained a mutation in this gene: one non-sense mutation deleting the last five amino acids of the protein and one in the splice donor site of exon 4 (Supplementary Table S1, Supplementary Figure S1). The *hei10(s)174* and *hei10(s)170* mutations were shown to be allelic, confirming that mutations in *At1g78790* cause the fertility restoration of *zmm* mutants.

This gene encodes a protein with high similarity with mammalian *MHF2*, and reciprocal Basic Local Alignment Search Tool (BLAST) analyses showed that *At1g78790* encodes the single *MHF2* homologue in the *Arabidopsis* genome (Supplementary Figure S1). We then named *At1g78790*, *AtMHF2*, and the three mutations *Atmhf2-1* (*zip4(s)2*), *Atmhf2-2* (*hei10(s)174*) *and Atmhf2-3* (*hei10(s)170*) (Supplementary Table S1). Human MHF1 and MHF2 were recently identified as a heterotetramer promoting FANCM activity and participating in somatic DNA damage repair and genome maintenance (10,11). Further, MHF1 and MHF2 have been shown to direct meiotic recombination outcome to NCOs in fission yeast (7).

**Figure 1.**
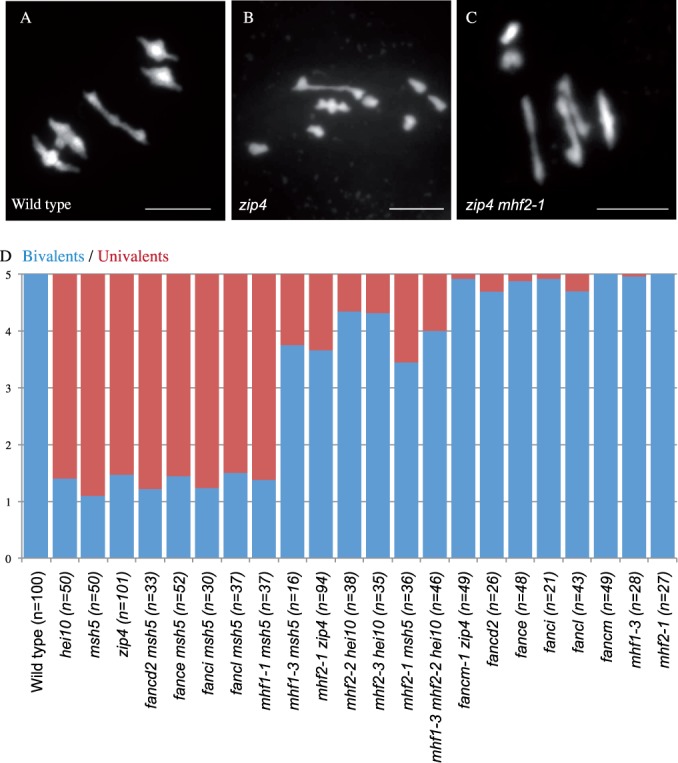
Bivalent formation analysis at metaphase I. (**A–C**) Metaphase I chromosome spreads of male meiocytes in three representative genotypes (A) wild type, (B) *Atzip4* (C) *Atzip4 Atmhf2-1*. Scale bar = 5 μm. (**D**) Average number of bivalents (blue) and pairs of univalents (red) per male meiocyte at metaphase I. Number of cells analysed is indicated in parentheses. *fancm zip4* and *zip4* data are from (6).

In the three suppressors with mutations in *AtMHF2*, chromosome spreads were performed to assess the level of bivalent formation. This showed that the restored fertility was indeed associated with increased bivalent formation at metaphase I compared to their *zmm* counterpart (Figure 1), suggesting that MHF2 has an anti-CO activity at meiosis in *Arabidopsis*. The restoration of bivalent formation was not complete, the *zmm* mutants, *zmm mhf2* double mutants and wild type having ∼1, ∼4 and 5 bivalent pairs, respectively (Figure 1). In contrast, *Atfancm* mutation almost completely restored bivalent formation of *zmm* mutants (4.9 bivalent pairs) suggesting that mutating *AtMHF2* has a lesser anti-CO effect than mutating *FANCM* at meiosis (Figure 1).

**Figure 2.**
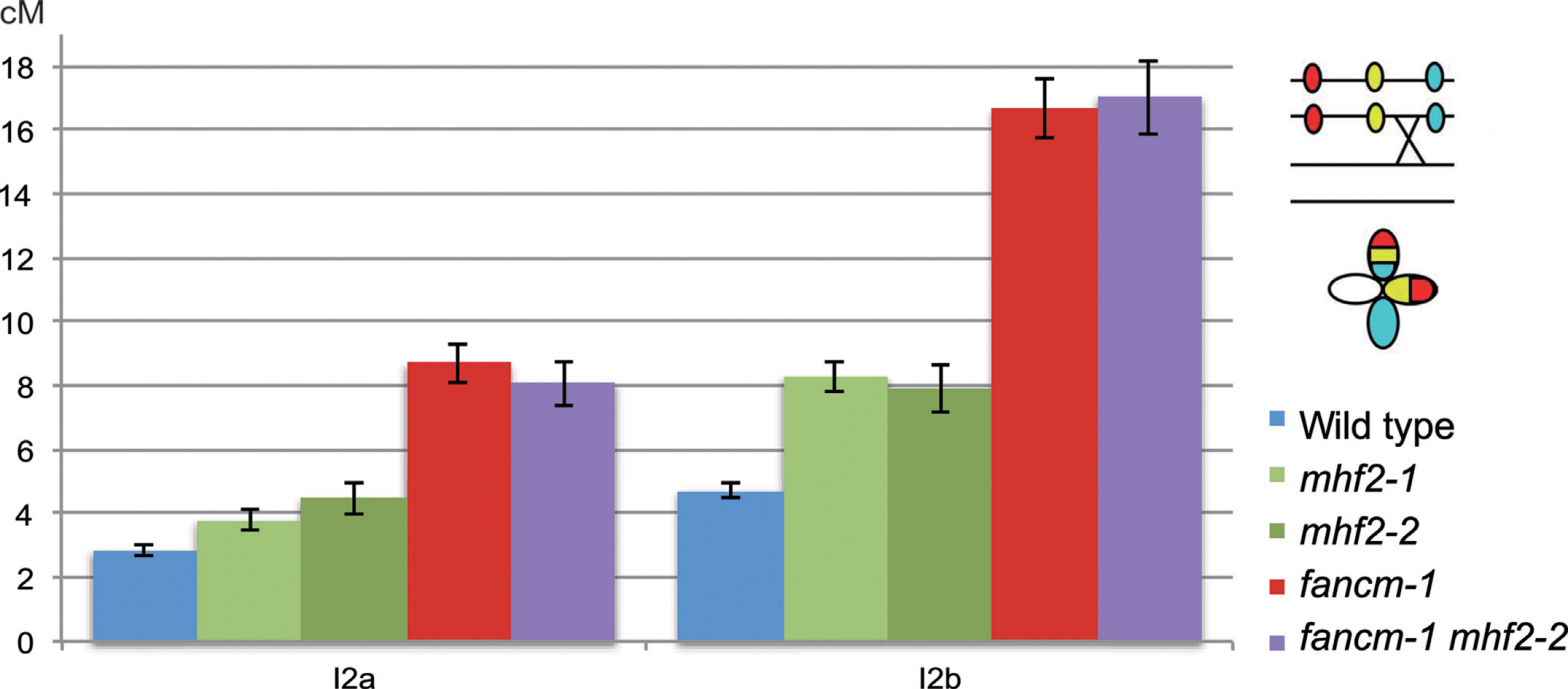
Genetic distances (cM) are increased in *mhf2* mutants. Genetic distances in two adjacent intervals on chromosome 2 using FTLs (19) were calculated with the Perkins equation (23) and are given in centiMorgans (cM). Error bars indicate standard deviation (± SD). Raw data and calculation can be found in Supplementary Table S2. One tetrad example and its interpretation are shown on the top right corner.

In the single *Atmhf2* mutants, metaphase I was indistinguishable from wild type with five bivalents (Figure 1, Supplementary Figure S2). Meiotic CO frequency in *Atmhf2* was then measured genetically using pollen tetrad analysis (19,22,23) (Figure 2, Supplementary Table S2). In the single mutants *Atmhf2-2* and *Atmhf2-1* map distances increased by ∼60% compared to wild type on the two intervals tested (Z-test, *P* < 6×10^−3^), demonstrating that MHF2 is a CO-limiting factor. This increase, while significant, is lower that what is observed in *fancm* (*P* < 10^−6^), further supporting the conclusion that *AtFANCM* is a more effective barrier to CO formation than *AtMHF2.*

### Mutations in MHF1, but not FANCL, FANCE, FANCI nor FANCD2 restore CO formation in *zmm* mutants

The identification of *AtMHF2* (this study) and *AtFANCM* (6) as factors limiting CO formation in *Arabidopsis*, prompted us to test for a similar role of other FA proteins. First, we examined the conservation of FA proteins among a selection of eukaryotes through reciprocal BLAST analysis and literature survey (6–8,10,11,24-60) (Table 1). The FA proteins known in humans can be categorized into three classes, according to the cascade of events in the process of repairing blocked replication forks: the FA core complex, the FA-ID complex and the downstream factors (8,10,11). Many FA proteins are conserved in animals and plants, suggesting that the pathway may be conserved in these kingdoms, making *Arabidopsis* a suitable model to address the function of FA factors. In contrast, fungi seem to lack the majority of FA components. In *Arabidopsis*, unique homologues of components of the core complex (in addition to FANCM and MHF2) were identified (FANC -E, -L, MHF1), as well as all three FA-ID complex members (-D2, -I, FAN1). Among the downstream genes two unique homologues were identified (-D1, -O), and a third possesses two homologues organized as a tandem duplication (-J). No putative homologues were found for the other FA members (Table 1). For MHF1, two genes predictions corresponding to the same locus are present in the *Arabidopsis* databases: AT5E46180 (61) and AT5G50930.1 (62) but it has recently been shown that only the transcript corresponding to AT5E46180 exists *in vivo* (63).

**Table 1.** Conservation of FA proteins among a selection of eukaryotes

	*H. sapiens*	*A. thaliana*	*S. cerevisiae*	*S. pombe*	*D. melanogaster*	*C. elegans*
FA core complex	FANCA (8)	-	-	-	-	-
	FANCB (24)	-	-	-	-	-
	FANCC (8)	-	-	-	-	-
	FANCE (8)	FANCE (At4g29560)	-	-	-	-
	FANCF (25)	-	-	-	-	-
	FANCG/XRCC9 (8)	-	-	-	-	-
	FANCL (8)	FANCL (At5g65740) (26)	-	-	FANCL (27)^a^	-
	FANCM/FAAP250 (8)	FANCM (At1g35530) (6,28)^a^	Mph1 (29,30)^a^	Fml1 (7,31)	FANCM (27)	FANCM-1/DRH3 (32,33)^a^
	MHF1/CENP-S/FAAP16 (10,11)	MHF1 (At5g50930) (34)	Mhf1 (10)^a^	Mhf1 (7)^a^	-	MHF1 (Y48E1C.1)
	MHF2/CENP-X/FAAP10 (10,11)	MHF2 (At1g78790)	Mhf2 (10)^a^	Mhf2 (7)^a^	-	MHF2 (F35H10.5)
	FAAP20 (35)	-	-	-	-	-
	FAAP24 (36)	-	-	-	-	-
	FAAP100 (37)	-	-	-	-	-
						-
				Fml1		
				Mhf1	-	
				Mhf2	-	
						
						
						
						
FA-ID and FAN1	FANCI (8)	FANCI (At5g49110)	-	-	FANCI	FANCI-1 (32,33)^a^
	FANCD2 (8)	FANCD2 (At4g14970) (26)	-	-	FANCD2 (27)^a^	FACD-2 (32–33,38–39)^a^
	FAN1 (8)	FAN1 (At1g48360)	-	Fan1 (40)^a^	-	FAN-1 (41–43)^a^
						
FA downstream partners	FANCD1/BRCA2 (8)	FANCD1 (At5g01630 & At4g00020) (64)^a^	-	-	BRCA2 (44)^a^	BRC-2 (33,45)^a^
	FANCJ/BRIP1/ BACH1 (8)	FANCJ (At1g20720 & At1g20750) (46)	-	-	-	DOG-1 (47)^a^
	FANCN/PALB2 (8)	-	-	-	-	-
	FANCO/RAD51C (8)	FANCO/RAD51C (At2g45280) (48,65–66)^a^	-	-	Spindle D (49)^a^	RFS-1/RAD51C (50)^a^
	FANCP/SLX4/ BTBD12 (51)	-	Slx4 (52)^a^	Slx4 (53)^a^	MUS312 (54,55)^a^	HIM-18/SLX4 (56)^a^
	FANCQ/ERCC4/ XPF/RAD1 (57)	FANCQ/RAD1 (At5g41150) (58,67)^a^
			Rad1 (59)^a^	Rad16 (60)^a^	MEI9 (55)^a^	XPF (56)^a^

Experimentally tested and putative homologues based on sequence similarity are shown.

The “-” symbol indicates no gene encoding protein with significant similarity was found.

^a^Experimental evidence of a role in DNA repair.

We analysed mutant lines in the three members of the core complex (*AtMHF1*, *AtFANCE*, *AtFANCL*) and two members of the FA-ID complex (*AtFANCD2*, *AtFANCI)* (see Materials and Methods).

For each targeted gene, we identified a T-DNA insertion that disrupts the genomic coding sequence (Supplementary Figure S3). We did not analyse the downstream partners AtFANCD1/BRCA2 and AtFANCO/RAD51C because they are essential for the repair of meiotic recombination intermediates in *Arabidopsis* and CO formation (64–66), and are therefore unlikely candidates for a role in limiting meiotic COs that could be detected. We did not analyse *FANCJ* either, because it is present as a tandem duplication, making its mutation unrealistic to obtain. None of the tested lines showed any obvious somatic defects. Meiosis was indistinguishable from wild type on chromosome spreads in *Atmhf1*, as shown above for *Atmhf2* (Supplementary Figure S2). This contrasts with the situation in fission yeast where MHF1/CENP-S and MHF2/CENP-X are required for balanced segregation of chromosomes at meiosis, through the establishment of proper kinetochore function, independently of FANCM and recombination (68). However a low frequency of univalents was detected in *Atfancd2*, *Atfance*, *Atfanci* and *Atfancl*, suggesting that these genes may have a minor role in promoting CO formation (Figure 1D and Supplementary Figure S2).

To assess the putative anti-CO activity of these genes we tested if their mutation could suppress the *zmm* lack of bivalents, as do the mutations in the genes *FANCM* and *MHF2*. For each T-DNA line, we obtained a double mutant with either *Atmsh5* or *Athei10* (15,18). None of the mutations of *AtFANCE, AtFANCL, AtFANCD2*, *AtFANCI* (Figure 1) nor *AtFANCQ/RAD1* (67) increased the number of bivalent in a *zmm* background. Even if we cannot formally exclude that some activity could be retained in the T-DNA mutants (although it appears unlikely in view of the positions of the 4.5 kb T-DNA insertions; Supplementary Figure S3), these results suggest that these FA genes do not have any anti-CO activity like FANCM and MHF2. In contrast, the double mutant *Atmhf1-*3 *Atmsh5* showed a large increase of bivalent formation compared to *Atmsh5* (Figure 1), showing that *At*MHF1 possesses a meiotic anti-CO function. The *Salk_119435* insertion (*mhf1-1* in (63)), which is inserted 41 base pairs in 3′ of the ATG was unable to restore bivalent formation of *Atmsh5*. This suggests that a functional MHF1 protein is produced at meiosis in this line.

The effect of *At*MHF1 depletion at meiosis was similar to that of *AtMHF2*, and thus weaker than that of FANCM, suggesting that like MHF2, MHF1 is a less efficient barrier to CO formation than FANCM. In summary, these data suggest that only a subset of the FA associated proteins, namely FANCM, MHF1 and MHF2, are involved in limiting meiotic COs.

### MHF1, MHF2 and FANCM act in the same pathway to limit meiotic COs

**Figure 3.**
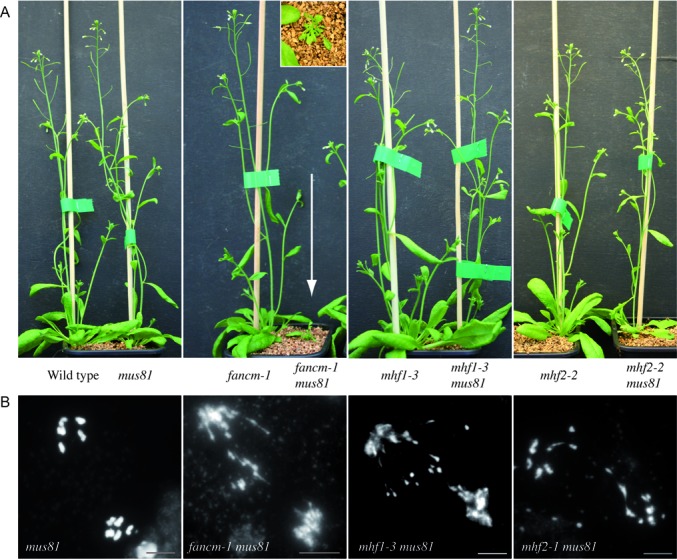
Genetic interaction of *Atmhf1*, *Atmhf2* and *Atfancm* with *Atmus81.* (**A**) Six weeks old plants are shown with the corresponding genotype indicated below. The arrow points to the sick *Atfancm Atmus81* double mutant for which an enlargement (top view) is shown. (**B**) Anaphase I chromosome spreads. Chromosome fragmentation can be observed in *Atfancm-1 Atmus81, Atmhf1–3 Atmus81* and *Atmhf2-1 Atmus81.* Scale bar = 5 μm.

We then tested whether *AtMHF1*, *AtMHF2* and *AtFANCM* act in the same pathway to limit meiotic COs. First, a triple mutant *hei10 mhf1 mhf2* showed the same level of bivalent formation compared to *msh5 mhf1, msh5 mhf2*, or *hei10 mhf2* (Figure 1D), suggesting that *AtMHF1* and *AtMHF2* act in the same pathway. As *fancm* mutation restores bivalent formation of *zmm* mutants to near wild-type levels, it cannot be tested if bivalent formation can be restored further in combination with *mhf1*or *mhf2*. This limitation can be overcome by measuring genetic CO formation. We therefore tested the effect of mutating both *AtFANCM* and *AtMHF2*, using tetrad analysis on one pair of adjacent intervals (I2a/I2b) (Figure 3). The genetic distances in *Atfancm Atmhf2-2* double mutant was higher than wild type (Z-test, *P* < 10^−5^) and *Atmhf2-2* (Z-test, *P* < 10^−4^), but not different from *Atfancm* (Z-test, *P* > 0.05), demonstrating that *AtFANCM* and *AtMHF2* limit COs in the same genetic pathway. This predicts that the extra COs in an *Atmhf2* mutant would arise from the class II pathway, as described for *Atfancm* (6). Consistently, the number of MLH1 foci per cell, a marker of class I, ZMM-dependent COs, are unchanged in *Atmhf2-1* compared to wild type [9.2±1.7 (*n* = 21) and 8.9±1.4 (*n* = 16), T-test *P* = 0.54] (Supplementary Figure S4). Further, as class II COs do not display interference, interference should be impaired in *Atmhf2* mutants. We used the tetrad data set to analyse interference through the calculation of the interference ratio (IR) (Supplementary Table S2). IR measures the effect of having recombination in one interval on the genetic distance of the adjacent interval. IR is close to 0 when having COs in one interval prevents CO formation in the adjacent interval, thus indicating positive interference; IR = 1 when interference is absent (22). Interference was detected in wild-type (IR I2b/I2a = 0.37; Z test *P*(IR = 1) = 1.2 10^−6^) but was undetectable in *Atmhf2-1* (IR I2b/I2a = 0.89; *P*(IR = 1) = 0.6) and *Atmhf2-2* (IR I2b/I2a = 1.02; *P*(IR = 1) = 0.9) (Supplementary Table S2C). Finally, as MUS81 promotes class II COs, we produced *Atmhf1-3 Atmus81* and *Atmhf2-1 Atmus81* double mutants (Figure 3). In these double mutants, chromosome fragmentation was observed at anaphase I, while this is not the case for the respective single mutants; *Atmhf1*, *Atmhf2* and *Atmus81* (Figure 3B). This shows that in absence of MHF1 or MHF2, MUS81 becomes necessary for efficient repair of DNA DSBs. This is reminiscent of the *Atfancm Atmus81* meiotic defects (6). Altogether this confirms that MHF1 and MHF2 act in the same pathway of FANCM to restrain class II meiotic CO. However, based on the partial restoration of bivalent formation in a *zmm* context (Figure 1) and on the measurement of recombination levels (Figure 2), it appears that MHF1 and MHF2 have a less prominent role than FANCM in limiting COs. While *Atfancm-1 Atmus81* plants are barely viable (6), growth and development of *Atmhf1-3 Atmus81* and *Atmhf2-1 Atmus81* plants did not have the same synthetic growth defect (Figure 3) until they enter into the reproductive phase and have reduced fertility. Similar results were reported by Dangel and colleagues (63). This suggests that MHF1 and MHF2 have a less important role than FANCM in the repair of somatic DNA damage, as they have a less important role in limiting meiotic COs. Similarly, in human HeLa cells, the absence of MHF1 or MHF2 leads to less severe genotoxic agent sensitivity than the absence of FANCM (10). Further, the MHF1 and MHF2 form a heterotetramer that enhances FANCM DNA binding and DNA branch migration activity *in vitro* but FANCM alone retains some activity independently of these two co-factors (10,11,69,70). We thus propose that during meiosis, MHF1 and MHF2 support the FANCM helicase anti-CO activity, but that FANCM is able to function partially in the absence of MHF1/MHF2. The other conserved members of the FA pathway, including the members of the core complex, do not seem to play a role in the FANCM-MHFs anti-CO activity. It has been previously suggested that FANCM, in addition to being a core component of the FA pathway, also has a function in somatic DNA repair independently of the FA pathway (discussed in (71)). Here we showed that the FANCM-MHF1-MHF2 module ensures a specific function as a barrier to CO formation in meiosis.

## SUPPLEMENTARY DATA

Supplementary Data are available at NAR Online.

SUPPLEMENTARY DATA

## FUNDINGS

European Community's Seventh Framework Programme FP7/2007-2013 [KBBE-2009-222883 (MeioSys); ERC 2011 StG 281659 (MeioSight)]. Funding for open access charge: European Community's Seventh Framework Programme FP7/2007-2013 [KBBE-2009-222883 (MeioSys); ERC 2011 StG 281659 (MeioSight)].
