# Supplementary material for: FANCM-associated proteins MHF1 and MHF2, but not the other Fanconi anemia factors, limit meiotic crossovers
Source: Nucleic Acids Res. 2014 Jul 18;42(14):9087–95. doi: 10.1093/nar/gku614 (PMC4132730; doi:10.1093/nar/gku614)
Supplement: SUPPLEMENTARY DATA [file supp_42_14_9087__index.html]

FANCM-associated proteins MHF1 and MHF2, but not the other Fanconi anemia factors, limit meiotic crossovers — FANCM-associated proteins MHF1 and MHF2, but not the other Fanconi anemia factors, limit meiotic crossovers — SUPPLEMENTARY DATA 

# FANCM-associated proteins MHF1 and MHF2, but not the other Fanconi anemia factors, limit meiotic crossovers

## SUPPLEMENTARY DATA

**Files in this Data Supplement:**

- SUPPLEMENTARY DATA
