# Supplementary material for: FANCM-associated proteins MHF1 and MHF2, but not the other Fanconi anemia factors, limit meiotic crossovers
Source: Nucleic Acids Res. 2014 Jul 18;42(14):9087–95. doi: 10.1093/nar/gku614 (PMC4132730; doi:10.1093/nar/gku614)
Supplement: SUPPLEMENTARY DATA [file supp_gku614_nar-01337-x-2014-File005.pdf]

Table S1. ***MHF2*** **alleles found in the** ***zmm*** **suppressor screens.**

Alleles		Genomic position of the mutation	Change in DNA sequence	Change in protein sequence
zip4(s)2	*mhf2-1*	Chr2 : 29,624,529	C > T	Affects splicing acceptor site of exon 3
hei10(s)174	*mhf2-2*	Chr2 : 29,623,950	G > A	Q > STOP - deletion of the 5 last amino acids
hei10(s)170	*mhf2-3*	Chr2 : 29,624,034	C > T	Affects splicing acceptor site of exon 8

Table S2. Analysis of recombination using FTL tetrads.

Table A shows in each genotype the number of tetrads of each class observed according to the classification of Berchowitz and Copenhaver (19). The data have been obtained from sister plants, and the wild type and *fancm* figures have been pooled with the corresponding data (which were not significantly different from their respective genotypes) from (6) to increase statistical power. Table B shows genetic distance ± S.D (standard deviation) in the two adjacent intervals calculated according to Perkins equation (34). Table C shows Interference between the intervals is estimated by the interference ratio (19, 22).

A

**I2a I2b**	Total	A	B	C	D	E	F	G	H	I	J	K	L
Wild type	4481	3831	395	242	6	1	1	1	3	1	0	0	0
mhf2-1	1847	1441	268	115	2	3	6	8	3	0	0	1	0
mhf2-2	1092	856	140	82	1	1	4	3	3	1	1	0	0
fancm-1	1556	932	364	186	17	10	15	10	14	4	3	1	0
fancm-1 mhf2-2	1226	766	268	129	9	10	11	11	14	3	4	1	0

B

	I2a		I2b	
	cM	SD	cM	SD
Wild type	2.87	*0.18*	4.71	*0.24*
mhf2-1	3.79	*0.34*	8.28	*0.50*
mhf2-2	4.49	*0.50*	7.92	*0.75*
fancm-1	8.71	*0.62*	16.68	*0.94*
fancm-1 mhf2-2	8.08	*0.69*	17.05	*1.16*

C

	Wild type	*mhf2-1*	*mhf2-2*	*fancm-1*	*fancm-1 mhf2-2*
d(I2b) without CO in I2a	4.68	8.35	7.91	17.10	16.79
d(I2b) with CO in I2a	2.40	7.41	8.06	14.43	18.54
**Interference ratio**	**0.51**	**0.89**	**1.02**	**0.84**	**1.10**
p (IR=1)	1.18×10^{-6}	0.56	0.97	0.31	0.64

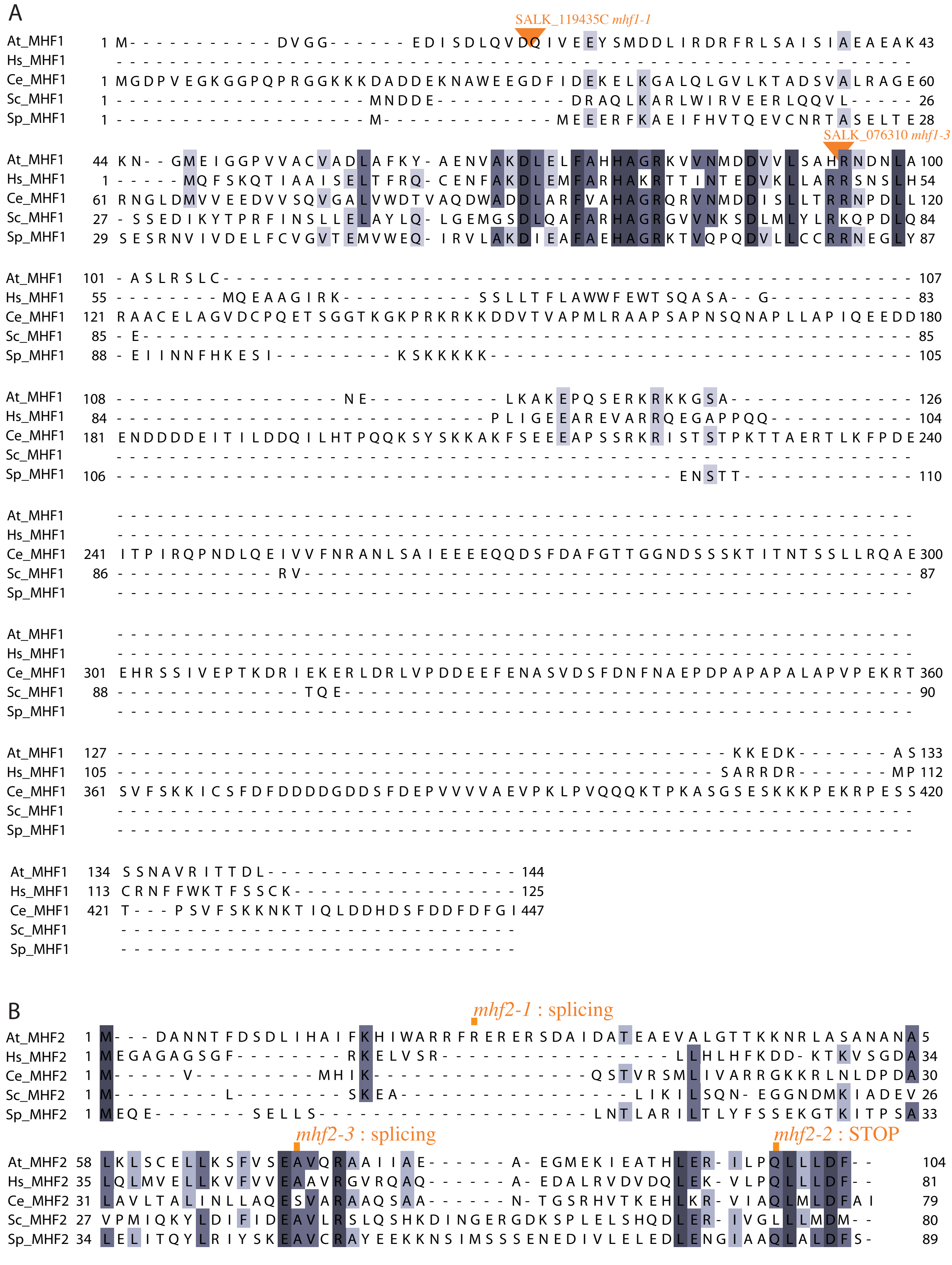

Figure S1. MHF1 and MHF2 protein alignments.

T-coffee protein alignment of MHF1 and MHF2 representative proteins. Position of MHF1 and MHF2 mutations studied here are indicated. Shading indicates 50% (light grey), 80% (medium grey) and 100% (dark grey) identity. *Hs: Homo sapiens, Ce: Caenorhabditis elegans, Sp: Schizosaccharomyces pombe, Sc: Saccharomyces cerevisiae. At: Arabidopsis thaliana.*

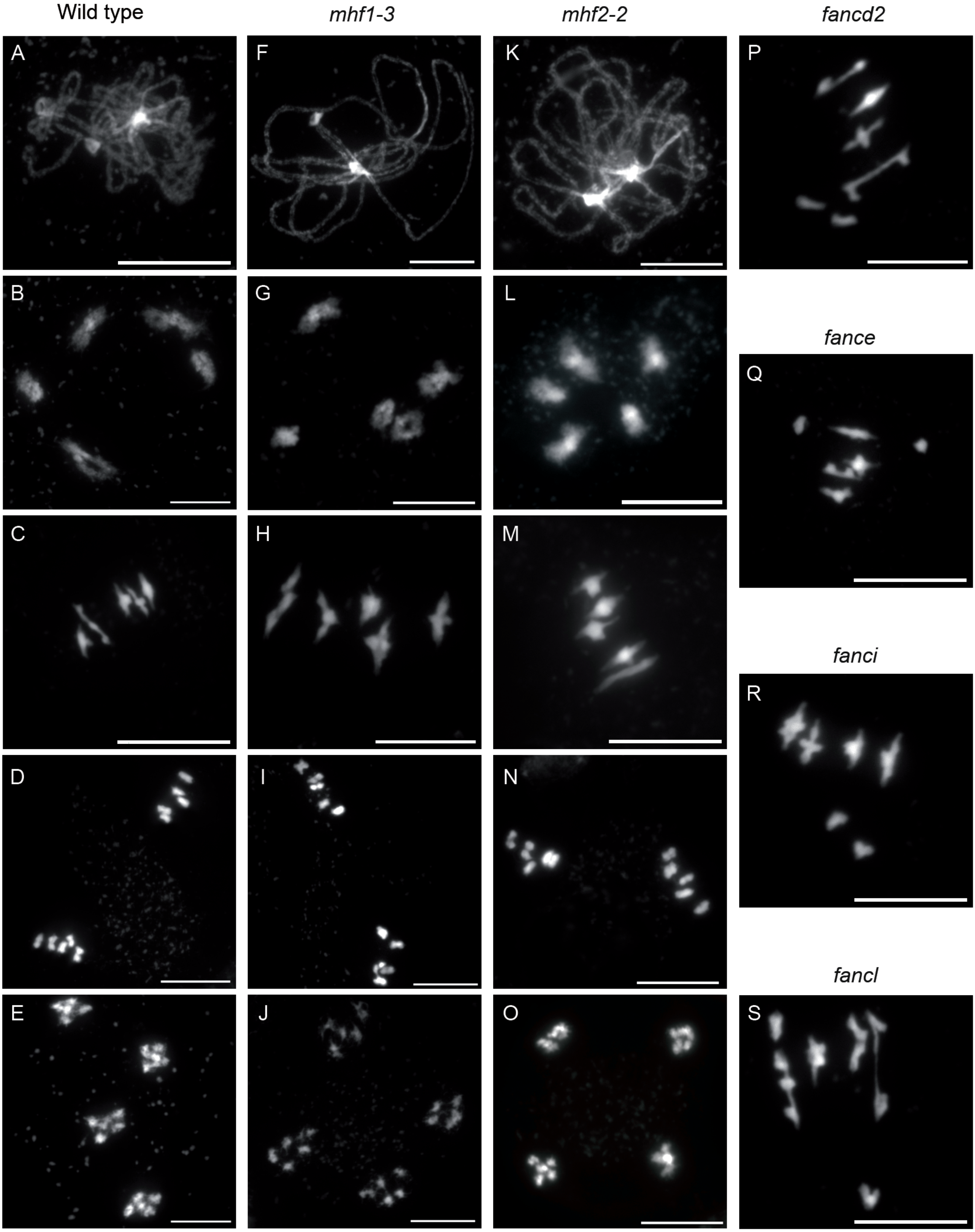

Figure S2. Male meiotic chromosome spreads in *mhf1-3 mhf2-1*, *fancd2, fance, fanci*, and *fancl*.
(A-E) Wild type. (F-J) *mhf1-3*. (K,O) *mhf2-1*. (P) *fancd2*. (Q) *fance*. (R) *fanci*. (S) *fancl*. (A, F, K) Pachytene. (B, G, L) diakinesis. (C, H, L, P, Q, R, S) Metaphase I. (D, I,N) Metaphase II. (E, J, O) Telophase II. Scale bar = 10μm.

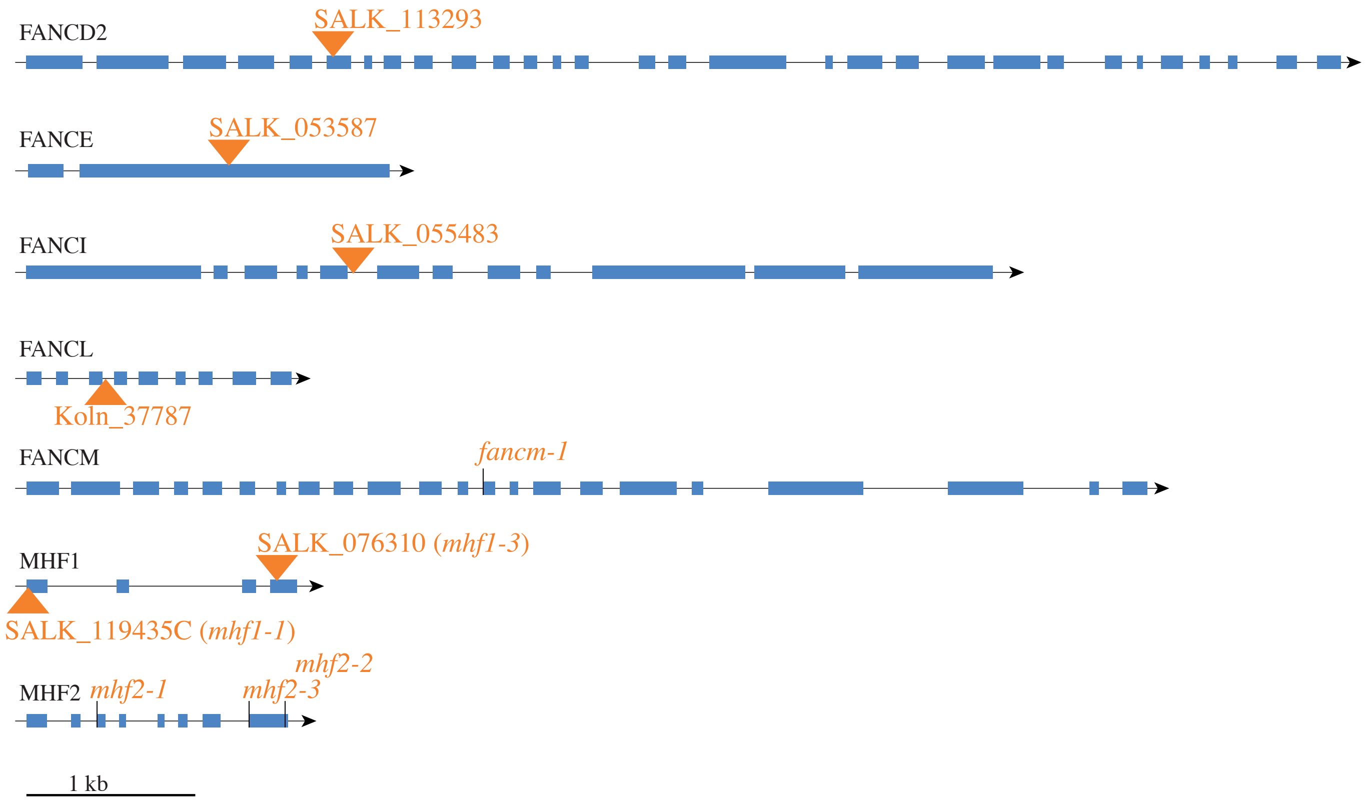

Figure S3. Structure of genes and position of mutations studied.
Exons are indicated as blue rectangles. Point mutations are indicated by vertical lines and T-DNA insertions are indicated with orange triangles

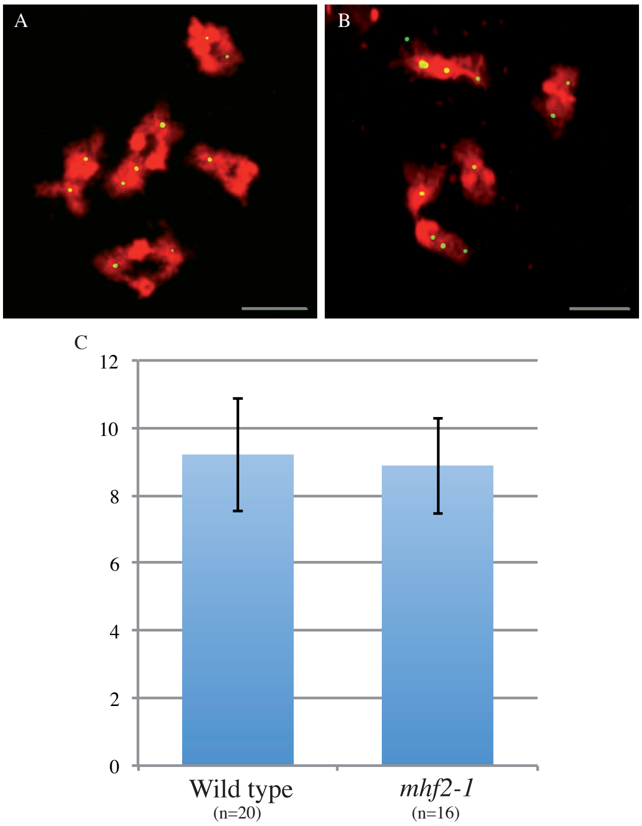

Figure S4. MLH1 immunolocalization.

Chromosome spreads of male meiocytes in (A) wild type and (B) mhf2-1. DNA stained by DAPI appears in red and MLH1 immuno-localization in green. Scale bar=5µm. (C) Quantification of MLH1 foci per cell in wild type (n=20) and *mhf2-1* (n=16). Error bars indicate standard deviation (± SD).

Expanded Methods

Mutdetect was developed to detect EMS mutations from whole genome resequencing experiment using paired-end libraries (Illumina technology).

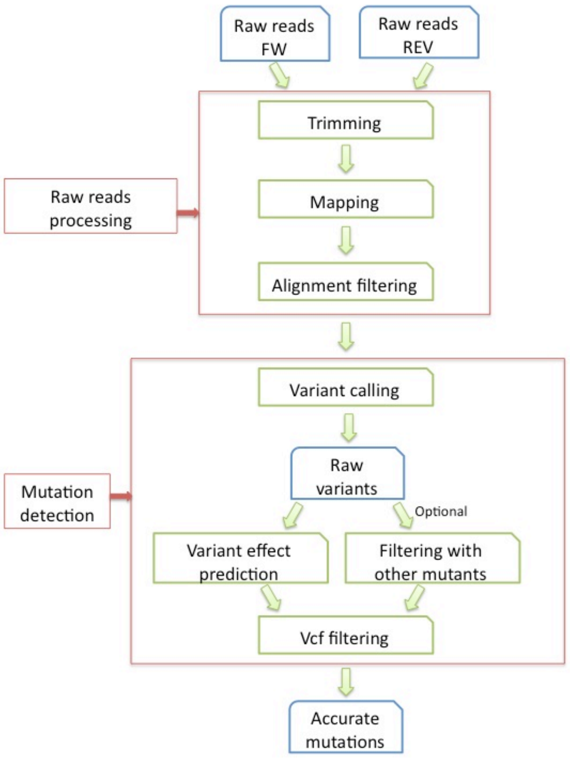

Raw reads processing

Sequences are cleaned using Trimmomatic [1] with a set of default parameters to trim reads in 5' and 3' if their base quality score is less than 20 and to cut reads when the mean quality in a 4 base window falls under 20. Trimmed sequences are kept if their length is at least 50 bp and if they remain paired.

Trimmed reads are aligned to the reference genome using BWA [2] with the following parameters [aln: -n 2 -o 1; sampe: –n 0 –N 0] to discard suboptimal hits. By default, multiple hits are allowed. Alignments are then filtered to allow a maximum of two independent events (substitution or micro-indel up to 5 bp) per read.

Mutation detection

Raw variants (SNPs and INDELS) are called from the filtered alignments by discarding bases with a quality score below 20 using the SAMtools suite [3]. To identify mutations that are specific to one line, Mutdetect offers the possibility to discard raw variants that are common with other raw variant files obtained from the corresponding wild type or from independent mutant(s). This allows the elimination of persisting mapping errors or genetic variations that may exist between the reference genome and the line before mutagenesis. SnpEFF [4] is used to predict the mutation effects (e.g. missence, splicing site, nonsense…).

The output table contains the identified mutations, their predicted effect and the descriptive statistics (e.g number of reads for each allele).

1. Bolger AM, Lohse M, Usadel B (2014) Trimmomatic: a flexible trimmer for Illumina sequence data. *bioinformatics (Oxford, England)*.
2. Li H, Durbin R (2009) Fast and accurate short read alignment with Burrows-Wheeler transform. *bioinformatics (Oxford, England)* **25**: 1754–1760.
3. Li H, Handsaker B, Wysoker A, Fennell T, Ruan J, Homer N, Marth G, Abecasis G, Durbin R, 1000 Genome Project Data Processing Subgroup (2009) The Sequence Alignment/Map format and SAMtools. *bioinformatics (Oxford, England)* **25**: 2078–2079.
4. Cingolani P, Platts A, Wang LL, Coon M, Nguyen T, Wang L, Land SJ, Lu X, Ruden DM (2012) A program for annotating and predicting the effects of single nucleotide polymorphisms, SnpEff: SNPs in the genome of Drosophila melanogaster strain w1118; iso-2; iso-3. *Fly (Austin)* **6**: 80–92.
